# Microchannel-assisted precision surgical treatment for thoracic ossification of the ligamentum flavum: a comparative retrospective study

**DOI:** 10.3389/fsurg.2026.1857299

**Published:** 2026-06-10

**Authors:** Zhi-Wei Wang, Ruo-Nan Liu, Na-Na Su, Dao-Kuo Liu, Chong Zhao, Chang-Kuan Li, Si-Bin Hu, Bao-Zhu Zhou

**Affiliations:** 1 Department of Spine Surgery, The Cangzhou Hospital of Integrated TCM-WM Hebei, Cangzhou, China; 2 Hebei Key Laboratory of Integrated Traditional and Western Medicine in Osteoarthrosis Research, The Cangzhou Hospital of Integrated TCM-WM Hebei, Cangzhou, China

**Keywords:** clinical effect, microchannel-assisted, minimally invasive, pain, TOLF

## Abstract

**Objectives:**

To investigate the clinical efficacy of the microchannel-assisted technique for precise treatment in patients with focal thoracic ossification of the ligamentum flavum.

**Methods:**

We retrospectively reviewed the clinical data of 38 patients diagnosed with focal thoracic ossification of the ligamentum flavum (TOLF) who underwent surgical treatment in the Department of Spine Surgery, The Cangzhou Hospital of Integrated TCM-WM Hebei, between January 2018 and December 2022. The patients were divided into two groups according to whether the microchannel-assisted technique was used during surgery: group A (microchannel-assisted group, *n* = 17) and group B (conventional open surgery group, *n* = 21). The surgery-related variables, radiological imaging results, and clinical outcomes were compared between the two groups.

**Results:**

None of the patients in either group experienced postoperative neurological deterioration. No statistically significant difference was observed in the JOA score improvement rate at the 2-year follow-up between the two groups (group A: 63.74 ± 18.55, group B: 61.56 ± 17.94, *P* > 0.05), indicating that the long-term efficacy of the microchannel-assisted technique for TOLF was comparable to that of conventional open surgery. No statistically significant differences were found between group A and group B regarding age, sex, BMI, smoking, alcohol consumption, heart disease, hypertension, and diabetes (*P* > 0.05). The VAS score for thoracic back pain in group A was superior to that in group B at 1 week and 1 month postoperatively (*P* < 0.05), but this difference was no longer observed at 3 months postoperatively. Statistically significant differences were observed between the two groups in operative time, blood loss, length of stay, wound length, and time to walking (*P* < 0.05). No statistically significant differences were found in the remaining relevant variables between the two groups (*P* > 0.05).

**Conclusions:**

This study confirms that the microchannel-assisted technique is a safe and effective minimally invasive treatment for focal thoracic ossification of the ligamentum flavum. This technique significantly reduces operative time, decreases intraoperative blood loss, promotes postoperative recovery, and lowers complication rates while ensuring comparable long-term neurological outcomes. These advantages are attributed to the concept of “precise decompression” and the preservation of paraspinal muscleoskeletal structures.

## Introduction

Thoracic ossification of the ligamentum flavum (TOLF) is a pathological process of heterotopic ossification that predominantly affects the lower thoracic spine. The ossified ligamentum flavum can cause severe spinal canal stenosis, resulting in significant neurological symptoms (1–4). TOLF is generally classified into focal, continuous, and skip types. The progression of TOLF is typically insidious; when clinical symptoms become apparent, the onset of overt symptoms often indicates a prolonged disease course and severe thoracic spinal cord compression. Surgical decompression remains the only effective treatment for TOLF. Conventional surgical approaches include laminoplasty, posterior laminectomy, and laminectomy with internal fixation (5, 6). However, these procedures are associated with considerable surgical trauma, substantial blood loss, and a high risk of intraoperative spinal cord injury, as well as postoperative drawbacks such as incisional pain, compromised spinal stability, and reduced mobility.

With the increasing use of the microchannel technique in the treatment of disc herniation and intraspinal tumors, its clinical value has become increasingly recognized. In recent years, this technique has gradually been extended to the treatment of patients with TOLF. This technique is designed to achieving precise decompression through a limited working channel, preserving the posterior tension band, improving visualization of the ossified ligament, and reducing the risk of dural tear, thereby minimizing disruption to spinal stability and facilitating perioperative recovery. However, reports on the use of this technique in focal TOLF remain limited. Therefore, this study aimed to investigate the clinical efficacy of the microchannel-assisted technique for precise treatment in patients with focal TOLF. This study hypothesizes that the microchannel-assisted technique for focal TOLF is non-inferior to conventional open surgery in terms of long-term neurological improvement, and has advantages in perioperative parameters.

## Materials and methods

### Inclusion of patients

We selected patients diagnosed with focal TOLF who underwent surgical treatment in the Department of Spine Surgery, The Cangzhou Hospital of Integrated TCM-WM Hebei, between January 2018 and December 2022. The inclusion criteria were as follows: (1) patients diagnosed with focal TOLF who underwent surgical treatment; (2) complete clinical data; (3) a minimum follow-up period of 2 years. Exclusion criteria included: (1) concomitant thoracic disc herniation or ossification of the posterior longitudinal ligament; (2) other concomitant spinal cord-related diseases; (3) trauma, inflammation, infection, or tumor involving the spine; (4) severe concomitant cervical or lumbar nerve compression; (5) continuous- or skip-type TOLF. Because the microchannel-assisted technique is more suitable for focal lesions, whereas continuous or skip lesions require more extensive decompression and are often combined with internal fixation, they are not applicable under the technical conditions of this study. A total of 38 patients were included in this study, comprising 21 males and 17 females. Based on the intraoperative use or non-use of the microchannel-assisted technique for non-randomized grouping, patients were consecutively enrolled with no randomization intervention and divided into Group A (microchannel-assisted group, *n* = 17) and Group B (conventional open surgery group, *n* = 21). Before the commencement of the study, the research protocol was submitted to and formally reviewed and approved by the Ethics Committee of The Cangzhou Hospital of Integrated TCM-WM Hebei. This approval confirmed that the study design and conduct strictly adhered to the ethical principles for human research established in the Declaration of Helsinki and complied with relevant national and international guidelines. The Ethics Committee waived the requirement for individual informed consent, because the retrospective and anonymous nature of the study posed no additional risk to patient privacy or rights.

### Study variables

Age, sex, BMI, smoking, alcohol consumption, heart disease, hypertension, diabetes, and preoperative thoracic back VAS scores were collected by different staff members at the time of admission. Patients were followed up at 1 week, 1 month, 3 months, 1 year, and 2 years postoperatively, and thoracic back VAS scores (0 indicates no pain and 10 indicates the worst imaginable pain) recorded at each time point. The analysis of operation-related variables, imaging results and clinical outcomes included the preoperative symptom duration, follow-up time, OLF segment, dural ossification, residual rate of cross-sectional spinal canal area on CT, shape on the sagittal MRI, operative time, blood loss, length of stay, wound length, time to walking, preoperative mJOA, postoperative mJOA, and JOA improvement rate. Furthermore, intraoperative and postoperative complications were analysed.

### Surgical methods

All 38 patients underwent surgery performed by the same surgical team. The 21 patients in group B underwent routine open laminectomy alone or laminectomy with internal fixation.

**Figure 1:**
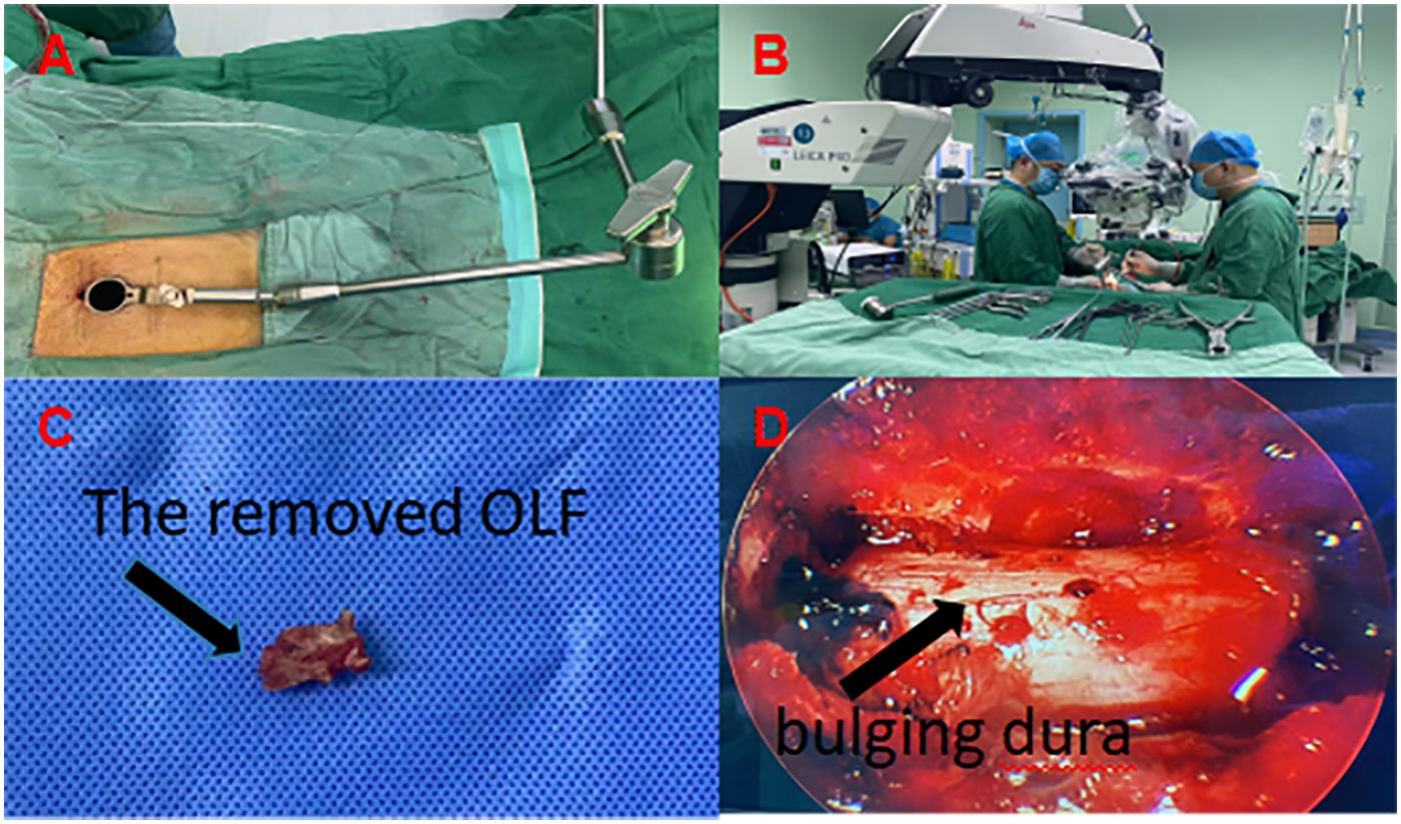
Intraoperative procedures. **(A)** shows the placement of the microchannel. **(B)** demonstrates the microscopic-assisted microsurgical manipulation. **(C)** presents the intraoperatively resected ossified ligamentum flavum. **(D)** reveals adequate dural bulging with good pulsation following sufficient decompression.

Group A: After induction of general anesthesia, the patient was placed in the prone position on the operating table. Intraoperative electromyography and somatosensory evoked potential monitoring were used. The operative segment was precisely localized using C-arm fluoroscopy. After routine disinfection and draping, a posterior midline incision of approximately 2–3 cm was made over the thoracic region. The skin and subcutaneous tissue were incised sequentially, and the muscle layer was gradually dilated using serial dilators. A microchannel retractor was then placed and secured. Under microscopic assistance, adequate decompression was achieved using a high-speed burr and Kerrison punch to remove the lamina and ossified ligamentum flavum When necessary, contralateral decompression was performed by resecting the contralateral lamina and ossified ligamentum flavum when necessary. For areas with dural adhesion or ossification, the periphery was circumferentially drilled, while thin bone fragments adherent to the dura in the central region were preserved, This approach achieved achieving effective circumferential decompression and allowing the spinal cord to “float,” with no residual dural sac compression and complete dural bulging with good pulsation (Figure 1). A gelatin sponge was placed over the decompression area, the incision was irrigated, andall instruments and gauze were counted. After confirming the absence of active bleeding, the incision was closed in layers, the skin was sutured, and a sterile dressing was applied. No drainage tube was placed intraoperatively in any patient in this group. Postoperatively, corticosteroids, dehydrating agents, and neurotrophic agents were administered as supportive treatment. Patients were allowed to ambulate without weight-bearing 1–2 days postoperatively and were discharged after incision healing.

Group B: The patient was placed in the prone position under general anaesthesia. During the operation, electromyography and somatosensory evoked potentials were monitored. The operative segment was confirmed by C-arm fluoroscopy. A posterior midline incision was made along the spinous process. The skin and subcutaneous tissue were dissected layer by layer, and the spinous process, lamina, and articular process were exposed. For patients who underwent laminectomy with instrumentation, pedicle screws were inserted first. Then, the spinous process and the outer layer of the lamina were removed, and the lateral laminae were removed by a high-speed grinding drill longitudinally using a high-speed drill along the inner edge of the bilateral facet joints. Then, the interlaminar space was enlarged, and the interspinous and ligamentous tissues between the adjacent vertebrae were removed. Then, a high-speed drill was used to thin the middle lamina under continuous normal saline irrigation, and with care taken to avoid excessive drilling until the ossified tissues were eggshell-thin and translucent. The remaining thin layer of bone was elevated with towel forceps, and the inner vertebral plate, OLF and dural mater were gently probed with a nerve dissector to assess the presence of adhesion. If no adhesion was present, the inner lamina and OLF were removed to achieve complete decompression. The surgeon continued resection bilaterally to the outer one-third of the facet joint until both sides of the dural sac were exposed; at this time, the dural sac was fully decompressed. If severe adhesion to the dura mater was present and could not be separated, the ossified tissue and dura were resected together, and the dural defect was repaired with local fascia. After decompression, in patients who underwent laminectomy with internal fixation, a pre-bent connecting rod was installed on the pedicle screws and then tightened and locked. Afterwards, the operative field was thoroughly irrigated, and an indwelling negative-pressure drainage tube was placed in any patient in this group, and the incision was sutured layer by layer. All patients received conventional postoperative management including dehydration agents and neurotrophic agents. Seven days after surgery, patients were encouraged to wear a thoracolumbar sacral orthosis while ambulating, and orthosis protection was maintained for approximately 3 months.

### Satisfaction evaluation

All patients were followed up in the outpatient clinic at 1 week, 1 month, 3 months, 1 year, and 2 years postoperatively. During follow-up assessments, thoracic back pain severity was evaluated using the VAS score, where 0 indicates “no pain” and 10 represents “the worst pain imaginable.” Patients were instructed to select a single number on the 11-point numerical scale (0–10) that best represented their current pain level. Neurological function was evaluated using the Japanese Orthopaedic Association (JOA) score during the follow-up. The thoracic neurological function JOA score (maximum, 11 points) was modified from the cervical JOA score (maximum, 17 points) (7, 8), 4 points for hand dexterity and 2 points for upper-limb sensation were removed based on the cervical JOA score. During follow-up, MRI and three-dimensional CTs were performed. The JOA improvement rate was calculated as follows: [(postoperative JOA score—preoperative JOA score)/(11—preoperative JOA score)] × 100%.

### Statistical analysis

All statistical analyses were performed using SPSS software version 22.0 (IBM, Armonk, NY, USA), and the significance level was *α* = 0.05. The continuous variables between the two groups were compared using independent-samples *t*-test or non-parametric test according to whether they were conformed to normal distribution and homogeneity of variance. Categorical variables were analyzed using the chi-square test. A *P* value < 0.05 was considered statistically significant.

## Results

All 38 patients (21 males and 17 females) underwent surgery successfully. All patients were followed up for a minimum of 2 years. The mean follow-up time at the final follow-up was 32.18 ± 7.17 months in group A and 30.58 ± 7.32 months in group B. The mean preoperative JOA score was 4.74 ± 1.44 in group A and 5.21 ± 1.51 in group B, while the mean JOA score at the final follow-up was 8.73 ± 1.28 and 8.51 ± 1.57, respectively. Both groups showed significant improvement in mean JOA scores at the final follow-up compared with preoperative values, with group A showing slightly higher scores than group B. No statistically significant difference was observed in the JOA score improvement rate at the 2-year follow-up between the two groups (group A: 63.74 ± 18.55, group B: 61.56 ± 17.94, *P* > 0.05), indicating that the long-term efficacy of the microchannel-assisted technique for TOLF was comparable to that of conventional open surgery. No statistically significant differences were found between group A and group B regarding age, sex, BMI, smoking, alcohol consumption, heart disease, hypertension and diabetes (*P* > 0.05) (Table 1). The VAS score for thoracic back pain in group A was lower than that in group B at 1 week (group A: 2.9 ± 0.7, group B: 4.3 ± 0.9) and 1 month (group A: 2.2 ± 1.1, group B: 2.8 ± 0.7) postoperatively (*P* < 0.05), but this difference was no longer observed at 3 months postoperatively. Statistically significant differences were observed between the two groups in operative time, blood loss, length of stay, wound length, and time to walking (*P* < 0.05). No statistically significant differences were found in the remaining variables between the two groups (*P* > 0.05) (Table 2).

**Table 1:** The main demographic variables of the patients with TOLF in 2 groups.

Characteristics	Group A (*n* = 17, 44.7%)	Group B (*n* = 21, 55.3%)	*P* value
Age (years)	58.47 ± 9.56	57.52 ± 6.44	0.612
Sex			0.796
Male	9 (52.9%)	12 (57.1%)	
Female	8 (47.1%)	9 (42.9%)	
BMI (kg/m²)			0.618
≤27	10 (58.8%)	14 (66.7%)	
>27	7 (41.2%)	7 (33.3%)	
Smoking			0.389
Yes	8 (47.1%)	7 (33.3%)	
No	9 (52.9%)	14 (66.7%)	
Drinking			0.917
Yes	10 (58.8%)	12 (57.1%)	
No	7 (41.2%)	9 (42.9%)	
Heart disease			0.984
Yes	4 (23.5%)	5 (23.8%)	
No	13 (76.5%)	16 (76.2%)	
Hypertension			0.255
Yes	5 (29.4%)	3 (14.3%)	
No	12 (70.6%)	18 (85.7%)	
Diabetes			0.912
Yes	3 (17.6%)	4 (19.1%)	
No	14 (82.4%)	17 (80.9%)	

BMI, Body Mass Index.

The main demographic variables in the two groups were not significantly.

Different (*P* > 0.05).

**Table 2:** The related risk factors of the patients with TOLF in two groups.

Characteristics	Group A (*n* = 17, 44.7%)	Group B (*n* = 21, 55.3%)	*P* value
Preoperative duration of symptoms (months)	15.71 ± 6.38	18.65 ± 9.08	0.326
Follow-up time (months)	32.18 ± 7.17	30.58 ± 7.32	0.381
OLF segment			0.922
Upper thoracic (T1-4)	2 (11.8%)	3 (14.3%)	
Middle thoracic (T5-8)	5 (29.4%)	7 (33.3%)	
Lower thoracic (T9-12)	10 (58.8%)	11 (52.4%)	
Dural ossification			0.819
Yes	2 (11.8%)	3 (14.3%)	
No	15 (88.2%)	18 (85.7%)	
Residual rate of cross-sectional spinal canal area on CT			0.252
≤60%	10 (58.8%)	16 (76.2%)	
>60%	7 (41.2%)	5 (23.8%)	
Shape on the sgittal MRI			0.275
Beak	14 (82.4%)	14 (66.7%)	
Round	3 (17.6%)	7 (33.3%)	
Operation time (min)	87 ± 22	103 ± 27	<0.001**
Blood loss (ml)	59.4 ± 27.6	171.2 ± 32.8	<0.001**
Length of stay (d)	5.8 ± 1.1	8.3 ± 1.7	<0.001**
Wound length (cm)	3.8 ± 0.4	10.2 ± 0.7	<0.001**
Time to walking (d)	2.2 ± 0.3	6.7 ± 0.5	<0.001**
Pre-mJOA	4.74 ± 1.44	5.21 ± 1.51	0.208
Post-mJOA (At last follow-up)	8.73 ± 1.28	8.51 ± 1.57	0.221
JOA improvement rate (At last follow-up)	63.74 ± 18.55	61.56 ± 17.94	0.263
Thoracic and back pain			
Pre-VAS	6.46 ± 0.75	7.21 ± 0.84	0.881
Post-VAS			
At 1 week	2.9 ± 0.7	4.3 ± 0.9	<0.001**
At 1 month	2.2 ± 1.1	2.8 ± 0.7	0.037*
At 3 month	1.6 ± 0.7	2.2 ± 0.6	0.161
At 1 year	1.2 ± 0.2	1.2 ± 0.3	0.328
At 2 year	1.1 ± 0.2	1.2 ± 0.3	0.261
Complications			
Wound infected	Nil	1 (4.7%)	
Delayed wound healing	Nil	1 (4.7%)	
Leakage of cerebrospinal fluid	Nil	2 (9.5%)	
Neurological deficit	Nil	Nil	
Intercostal pain	Nil	1 (4.7%)	
Thrombosis of lower extremities	Nil	2 (9.5%)	

mJOA:Modified Japanese Orthopaedic Association.

* The difference possessing statistical significance, *P* < 0.05.

** *P* = 0.000.

**Figure 2:**
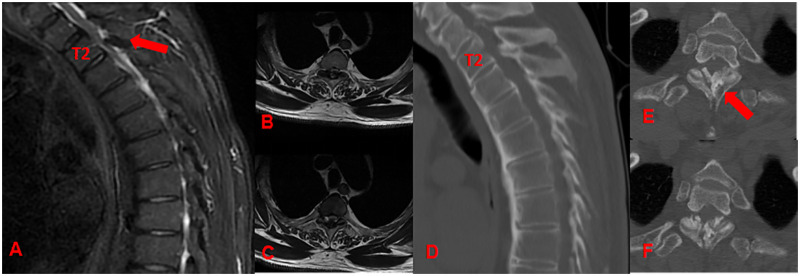
Preoperative CT and MRI images of a patient undergoing microscope-assisted surgery. **(A,B,C)** (preoperative sagittal and axial MRI scans) reveal hypertrophied ligamentum flavum and compressive myelopathy with associated spinal cord signal change. **(D,E,F)** (preoperative sagittal and axial CT scans with three-dimensional reconstruction) demonstrate severe OLF with significant compression of the spinal canal.

**Figure 3:**
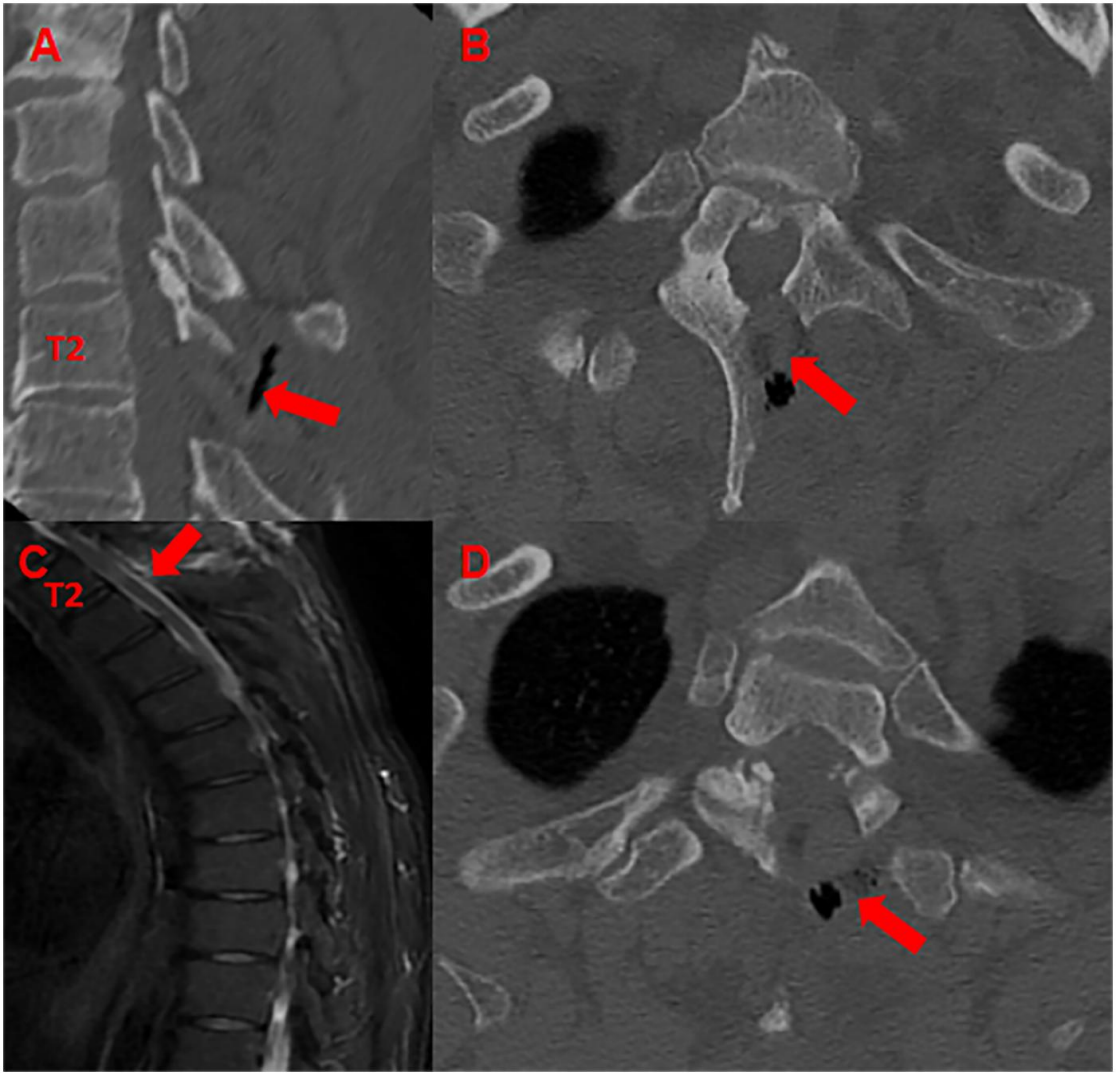
Postoperative CT and MRI images of a patient undergoing microscope-assisted surgery. **(A,B,D)** (postoperative sagittal and axial CT scans with three-dimensional reconstruction) demonstrate complete removal of the OLF, adequate decompression with a significantly enlarged spinal canal and increased cross-sectional area, successful bilateral neural element decompression, limited bone resection, and preserved facet joints. **(C)** (postoperative sagittal MRI scan) showing complete resolution of the spinal cord compression and full re-expansion of the thecal sac.

Furthermore, compared with group A, one patient in group B developed progressive worsening of bilateral lower-limb symptoms 12 h postoperatively, which was considered to be caused by an epidural hematoma. Following emergency debridement and administration of corticosteroids and dehydrating agents, the patient's muscle strength gradually recovered to the preoperative level at 1 month postoperatively. In group B, one patient developed postoperative incisional infection, and two patients developed a cerebrospinal fluid leakage. Compared with group B, group A had advantages including shorter operative time, less intraoperative blood loss, shorter hospital stay, earlier ambulation, fewer complications, and faster recovery (Figures 2, 3).

## Discussion

This single-center retrospective study systematically evaluated the clinical value of the microchannel-assisted technique in the surgical treatment of focal TOLF. The results showed that, compared with conventional open surgery, the microchannel-assisted technique not only achieved comparable long-term neurological improvement but also offered significant advantages in reducing perioperative trauma, accelerating functional recovery, and preventing complications.

In this study, there was no significant difference in the JOA score improvement rate between the two groups at 2 years postoperatively. This key finding suggests that the microchannel-assisted technique can achieve decompression outcomes equivalent to those of open surgery. This equivalence may be explained by the evolution of the surgical concept from traditional “adequate decompression” to modern “precise decompression.” The core principle of conventional open surgery is to ensure adequate decompression through extensive exposure and *en bloc* resection of bony structures. However, this extensive exposure strategy often requires wide paravertebral muscle dissection, disruption of the spinous process–lamina complex, and excessive resection of the facet joints, thereby increasing the risk of postoperative iatrogenic instability and axial pain (9–12). In contrast, the essence of the microchannel-assisted technique lies in “targeted decompression.” This approach involves preoperative precise localization of the ossified segments and the areas of greatest compression using CT and MRI, intraoperative accurate placement of the working channel into the target region under C-arm guidance, and use of the high-resolution microscopic field to clearly identify the three-dimensional anatomical relationships among the ossified ligamentum flavum, dural sac, and facet joints. This approach enables decompression through “marginal fenestration and piecemeal resection”, removing only the compressive ossified tissue while preserving the spinous process, contralateral lamina, and most of the facet joints. Thus, the structural integrity of the posterior spinal column can be preserved without compromising the adequacy of decompression. Extensive laminectomy has been reported to be positively associated with the progression of postoperative thoracic kyphotic angle (13, 14). Preservation of the spinous process, facet joints, and ligament attachments inherent to the microchannel-assisted technique helps maintains the posterior tension band of the spine, which may be important for maintaining long-term sagittal balance after thoracic surgery, reducing adjacent segment degeneration, and avoiding iatrogenic kyphotic deformity.

The VAS score for back pain in group A was significantly lower than that in group B at 1 week and 1 month postoperatively, and both the time to ambulation and length of hospital stay were significantly shorter. These differences may be primarily attributable to the muscle-sparing nature of the technique. Conventional open surgery requires a long midline incision along the spinous process (mean, 10.2 cm in group B in this study), together with extensive bilateral paravertebral muscle dissection to the lateral aspect of the facet joints. This process inevitably disrupts the attachment of the multifidus muscle on the spinous process and may damage its segmental innervation, leading to postoperative muscle ischemia, denervation, and scarring (9). In contrast, the microchannel technique uses sequential dilators to separate muscle fibers rather than transecting the paraspinal muscles. The working channel provides an operating window of approximately 2–3 cm in diameter, thereby maximizing preservation of the anatomical integrity and blood supply of the paravertebral muscles. This “muscle-sparing” approach may translates into reduced postoperative pain, decreased analgesic medications, and accelerated functional rehabilitation.

In this study, no surgery-related complications occurred in group A, whereas group B had one case of progressive worsening of bilateral lower-limb symptoms 12 h postoperatively, one case of incisional infection, and two cases of cerebrospinal fluid leakage. Cerebrospinal fluid leakage is one of the most challenging complications in TOLF surgery, according to the literature, its incidence in conventional open surgery ranges from 15% to 30% (15–18). This is primarily attributable to the dense adhesion between the ossified ligamentum flavum and the dural sac, with dural ossification occurring in some cases (16, 17). In conventional open surgery, limited visualization and insufficient magnification, it is often difficult for surgeons to clearly identify the interface between the ossified tissue and the dura, and forceful dissection may therefore result in dural tearing. In contrast, the microchannel technique provides coaxial illumination, high magnification, and a stereoscopic visual field, which is particularly beneficial for lateral or ventral ossified lesions, avoiding blind manipulation. These features allow the surgeon to gradually thin the ossified ligamentum flavum to a translucent state using an “eggshell-like drilling” technique, followed by gentle separation with a micro-dissector, thereby significantly reducing the risk of dural injury. In cases where the ossified tissue is completely fused with the dura, we adopted a strategy of “preserving a thin bony shell” to avoid forceful dissection, further enhance surgical safety. In addition to a clear surgical field, the microchannel-assisted technique employs an “eggshell-like drilling + preserving a thin bony shell” strategy to avoid forceful separation of dural adhesions, which is a key technical factor in reducing cerebrospinal fluid leakage. This finding is consistent with the JOA improvement rate (70.5%) reported by Zhu et al. (19) for channel-assisted surgery, further supporting the reliability of the precise decompression strategy in maintaining long-term efficacy.

This study provides supportive evidence for the minimally invasive treatment of focal TOLF, but certain limitations should be acknowledged. First, as a single-center retrospective study, the sample size was relatively small, and the non-randomized design may have introduced selection bias. Second, this study included only patients with focal TOLF, and the conclusions cannot be directly generalized to more complex cases involving multilevel disease, continuous-type TOLF, or concomitant ossification of the posterior longitudinal ligament. Third, This study did not perform MRI-based quantitative assessment of postoperative paraspinal muscle atrophy or fatty infiltration; therefore, the statement regarding“preservation of muscle anatomical integrity”is primarily based on intraoperative observation and inferences from previous literature. Future studies should include imaging indicators. For such patients, minimally invasive techniques should be applied with greater caution, and supplementary internal fixation may still be necessary to maintain spinal stability when indicated. In the future, large-scale, multicenter, prospective randomized controlled trials are warranted to further validate these findings and to define the scope of application of this technique in more complex cases.

## Conclusions

This study suggests that the microchannel-assisted technique is a safe and effective minimally invasive treatment for focal thoracic ossification of the ligamentum flavum. This technique significantly reduces operative time, decreases intraoperative blood loss, promotes postoperative recovery, and lowers complication rates while ensuring comparable long-term neurological outcomes. These advantages may be related to the concept of “precise decompression” and the preservation of paraspinal musculoskeletal structures. These findings support the application of the microchannel-assisted technique by experienced surgeons, but further validation in prospective, preferably randomized controlled, studies is warranted.

## Data Availability

The raw data supporting the conclusions of this article will be made available by the authors, without undue reservation.
